# Feasibility, safety and effectiveness of mapping system assisted conduction system pacing: a single-center prospective study

**DOI:** 10.1038/s41598-023-36546-x

**Published:** 2023-06-15

**Authors:** Liang Wang, Suxia Yang, Baopeng Tang, Feifei Wang, Wanyue Sang, Yafan Han, Lu Wang, Xianhui Zhou, Jianghua Zhang, Qiang Xing, Zukela Tuerhong, Jiasuoer Xiaokereti, Yankai Guo, Yaodong Li

**Affiliations:** 1 Department of Pacing and Electrophysiology, The First Affiliated Hospital of Xinjiang Medical University, Ürümqi, China; 2 Department of Cardiac Electrophysiology and Remodeling, The First Affiliated Hospital of Xinjiang Medical University, Ürümqi, Xinjiang China; 3 Xinjiang First Aid Center, People’s Hospital of Xinjiang Uygur Autonomous Region, Ürümqi, Xinjiang China

**Keywords:** Interventional cardiology, Randomized controlled trials

## Abstract

To assess pacing and electrophysiological parameters, as well as mid-term outcomes, among patients undergoing His bundle pacing (HBP) guided by KODEX-EPD (a novel mapping system). Consecutive patients undergoing conduction system pacing (CSP) for bradycardia indications were evaluated. Procedural and fluoroscopic times and pacing characteristics were compared between conventional fluoroscopy (the standard group, N = 20 cases) and KODEX-EPD mapping system guided group (the KODEX group, N = 20cases) at CSP implantation and all patients were followed at 6-month. HBP was achieved in all patients (the standard group 20/20 and the KODEX group 20/20). There was no difference in the mean procedure time between the two groups (63.7 ± 9.3 vs. 78.2 ± 25.1 min, *p* = 0.33). Compared with the standard group, the KODEX group significantly reduced the intraoperative X-ray exposure time (3.8 ± 0.5 vs. 19.3 ± 5.1 min, *p* < 0.05) and X-ray dose (23.6 ± 5.4 vs. 120.2 ± 38.3 mGy, *p* < 0.05). There were no significant differences in atrial impedance (643.0 ± 98.8 vs. 591.5 ± 92.1 Ω, *p* = 0.09), atrial sensing (2.9 ± 0.8 vs. 2.5 ± 0.8 mV, *p* = 0.08), ventricular sensing (12.8 ± 2.4 vs. 13.3 ± 3.3 mV, *p* = 0.63),atrial pacing threshold (1.0 ± 0.2 vs. 1.0 ± 0.1 V/0.4 ms, *p* = 0.81) and ventricular pacing threshold (1.0 ± 0.2 vs. 0.9 ± 0.1 V/0.4 ms, *p* = 0.63) between two groups, There were statistical differences in ventricular impedance (640.0 ± 80.3 vs. 702.0 ± 86.1 Ω, *p* < 0.05). There was no statistical significance in pacing parameters between the two groups at 6 months after procedure (*p* > 0.05). During the 6-months follow-up period, no adverse events occurred in the two groups. It can be concluded that KODEX-EPD can safely guide His bundle branch pacing lead implantation with reduced fluoroscopic time and dose without lengthening the procedure time.

## Introduction

Cardiac pacing is an effective therapy for bradycardia caused by cardiac conduction dysfunction. The pacing site at the right ventricular apex has been traditionally chosen because of easiness for placing a transvenous pacing lead. Multiple studies performed in the last two decades have shown the detrimental impact of permanent right ventricular pacing (RVP) on clinical outcomes due to ventricular mechanical dyssynchrony secondary to electrical dyssynchrony^1^. Thus, alternative pacing sites, such as His bundle pacing (HBP) which utilizes the natural cardiac conduction system, was introduced, and pacemaker therapy has entered a new era. However, HBP has challenging issues, including the difficulty in lead positioning and fixation, lengthy procedure time, high pacing threshold, long-term instability, and inability to deal with the block below the His bundle^2,3^. Left bundle branch pacing (LBBP) was initially described by Huang et al.^4^ in 2017 as a bail-out strategy after failure of left ventricular lead placement for HBP. Recent studies have shown that during the implantation of LBBP lead pacing impedance and sensing gradually decrease, and loss of injury current and a sharp decline in impedance and sensing frequently indicate ventricular septal perforation. It is important to pay attention to any postoperatively delayed ventricular septal perforation that could result in ventricular left-to-right shunting^5^. Till now, consensus criteria for LBBP capture are lacking and need to be better characterized. Some studies have shown that visualization by means of contrast injection can greatly shorten the procedure and fluoroscopic time (FT), but the overall FT is still relatively long^6,7^. Cardiac images provided by KODEX-EPD (3D elecroanatomical mapping system) may provide a reliable anatomical reference for defining the region of the His bundle branch area and the RV entry site, and thus has a potential to significantly reduce the fluoroscopic exposure^8,9^.

The KODEX-EPD mapping system, a new electrophysiological anatomical mapping and navigation technology (Philips, Netherlands), uses seven surface dielectric sensors and catheter electrodes to generate and receive electric fields at different frequencies, achieving accurate anatomical imaging and catheter positioning through global and local dielectric sensing technology. Global dielectric sensing is to generate a global electric field throughout the torso through a body surface dielectric sensor by using low frequency alternating current of many different frequencies. The electric field distribution in the heart is measured as the catheter electrode moves to locate the catheter and construct the anatomical structure. Local dielectric sensing is the application of a local electric field at a low frequency through the intracardiac catheter electrode to image local tissues, acquire additional anatomic information. The whole process is non-contact modeling, which distinguishes and displays the cardiac solution structure by detecting the gradient and bend of electric field.

In this article, forty (40) patients indicated for ventricular pacing therapy were enrolled in a randomized controlled study at a single center to examine the feasibility, safety and effectiveness of KODEX system guided CSP lead implantation in comparison to conventional CSP implantation fluoroscopy.

## Methods and data collection

### Patients

From August 2021 to April 2022, 40 consecutive patients who underwent permanent pacemaker implantation in the First Affiliated Hospital of Xinjiang Medical University were prospectively enrolled, and 40 patients were randomly divided into the conventional fluoroscopy group and KODEX group in a fashion of 1:1 according to random number table.All procedures were performed by the same team of physician who has experience in CSP implantation, with one operator to perform operations. The same operator performed the surgery to make sure it did not affect the results. Inclusion criteria were minimum age of 18 years with Class I and II indications for pacing^10^ who were suitable for HBP pacing and had the ability to complete the study follow-up. Exclusion criteria included: patients with CRT implantation, patients with coagulation disorders, patients with cardiac function which were above Grade III and unable to tolerate the procedure; patients who were complicated with hyperthyroidism, valvular heart disease or malignant tumor; patients who were complicated with lung, liver and renal dysfunction; patients with psychiatric illness, Cognitive Communication Disorders; patients with failure of an implanted pacemaker.

The current study, involving humans, was performed in accordance with the tenets of the Declaration of Helsinki. The study was approved by the ethics committee of The First Affiliated Hospital of Xinjiang Medical University (Approval Number, 202301-24). Informed consent was obtained from the participants.

### Methods

Routine examination including 12-lead electrocardiogram and echocardiography were performed to observe the ventricular anatomy structure, and antibiotics were prophylactically applied half an hour before procedure.

**Figure 1:**
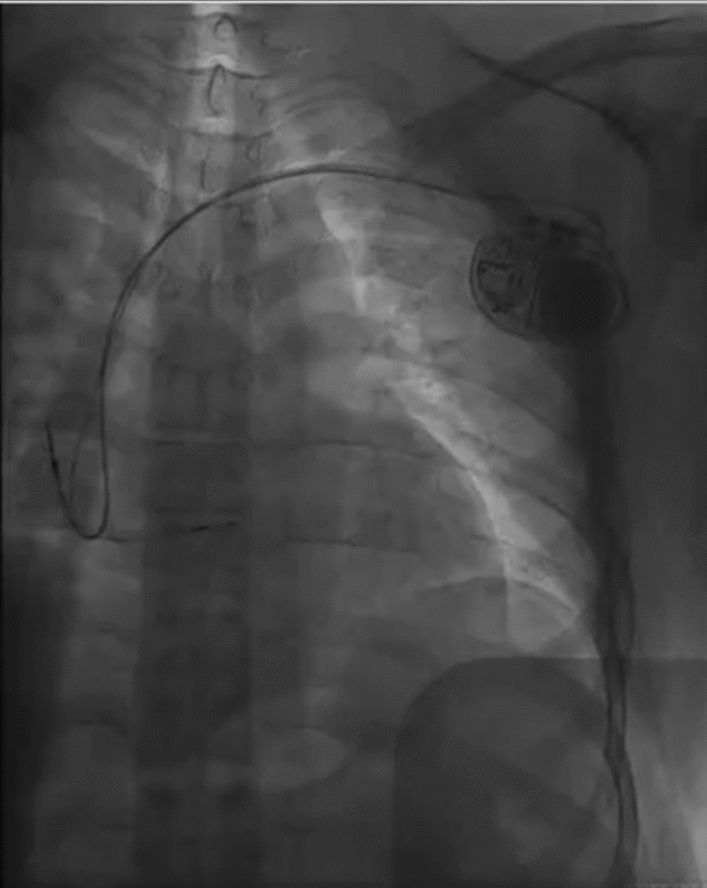
Final lead position in the fluoroscopic image in the standard group.

*The standard group* The patient was positioned supine with ECG monitoring and multi-conductive physiological apparatus connected. The 3830 lead and the C315His sheath are used as the pacing lead and the delivery catheter. Under the right anterior oblique 30° (RAO 30°) fluoroscopic view, a Model 3830 pacing lead (Medtronic) was placed at the atrioventricular node to determine the optimal location of HBP lead deployment. At this location the His potential was recorded, or HB captured by unipolar pace mapping (Fig. 1). The lead was fixed at a satisfactory position with respect to pacing parameters.

**Figure 2:**
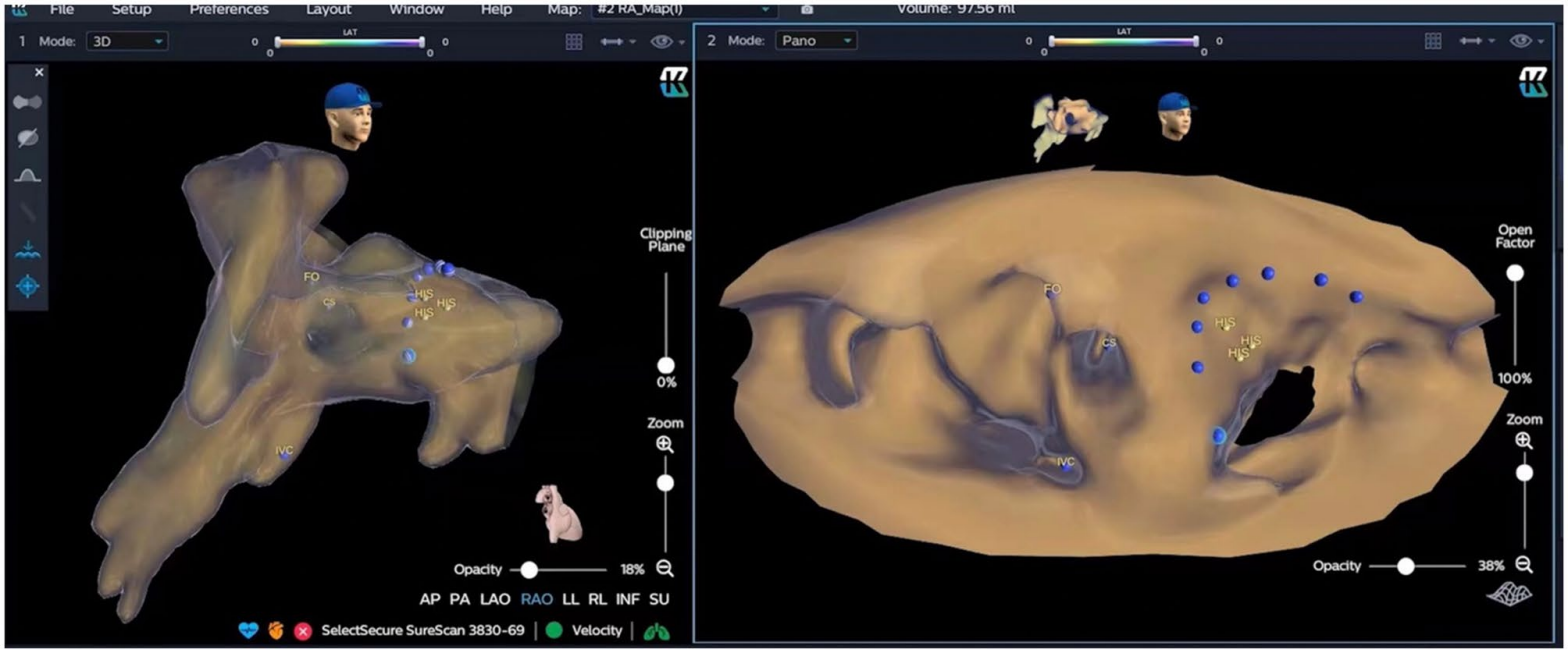
Position of HB region. KODEX-EPD system displayed anatomical images in three dimensions (on the left) and the anatomical structure of the cardiac chambers in a panoramic view (on the right). The yellow dots represent the sites where the HB potential can be recorded. The blue dots represent the path of the tricuspid valve annulus. *FO* fossa ovalis, *IVC*, inferior vena cava, *CS* coronary sinus.

**Figure 3:**
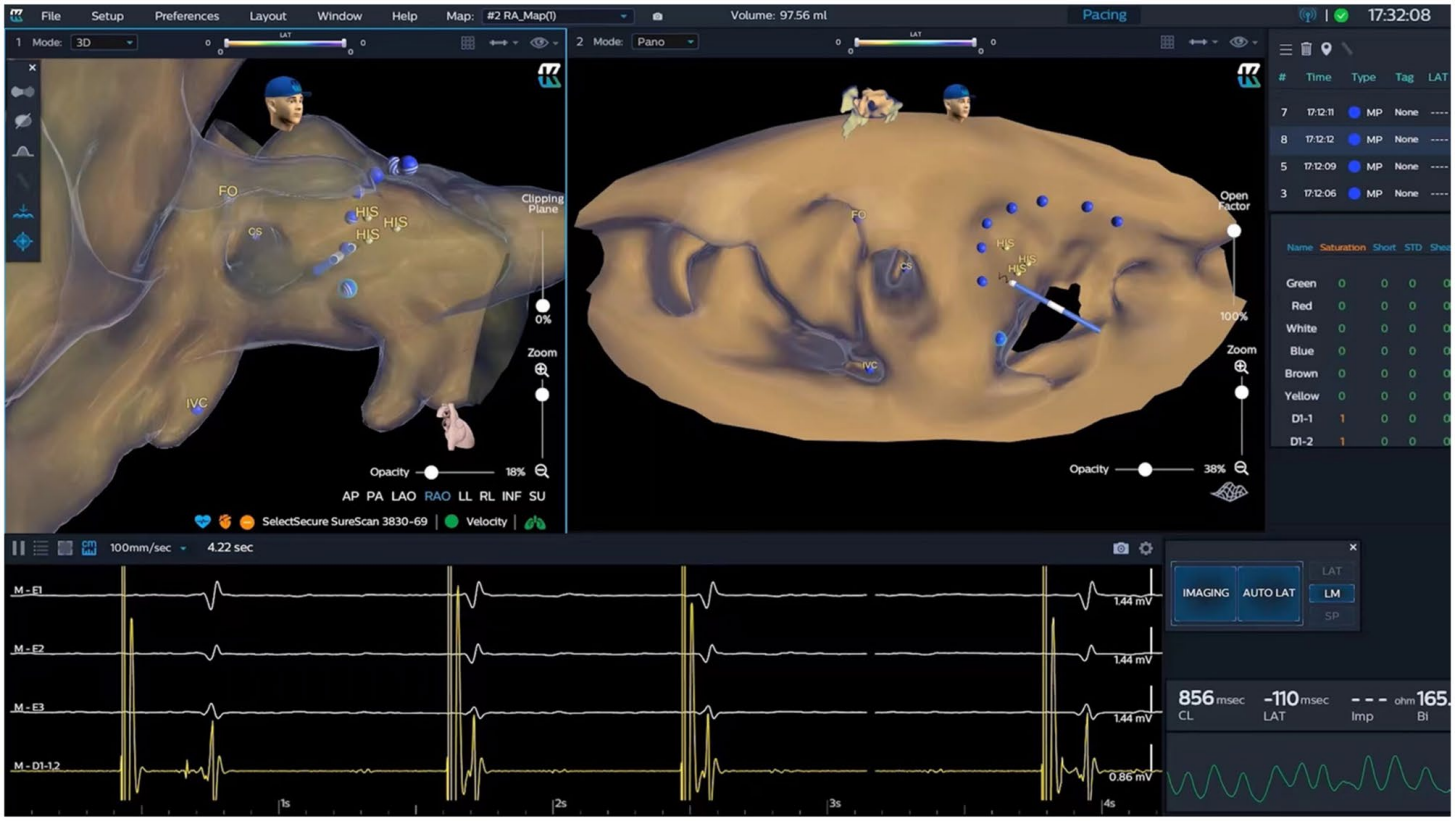
The KODEX-EPD mapping system showed three-dimensional anatomical image and the Panorama View (PANO View) of anatomical structure of cardiac chambers (Right): guided 3830 leads implantations.

**Figure 4:**
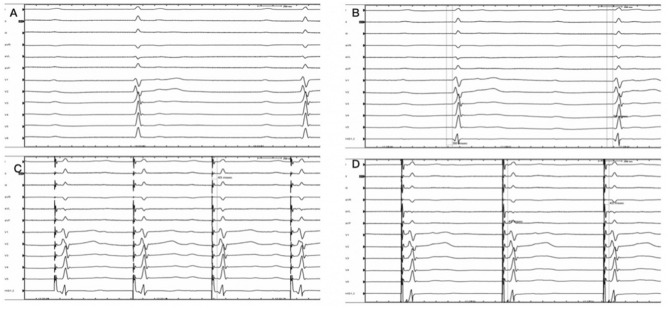
Intraoperative electrocardiogram (**A**) Preoperative electrocardiogram suggested third-degree atrioventricular block. (**B**) Intraoperative mapping prompt: the H–V was constant and there was a block above the His Bundle. (**C**) Intraoperative electrocardiogram: 1 V. (**D**) Intraoperative electrocardiogram: 5 V.

**Figure 5:**
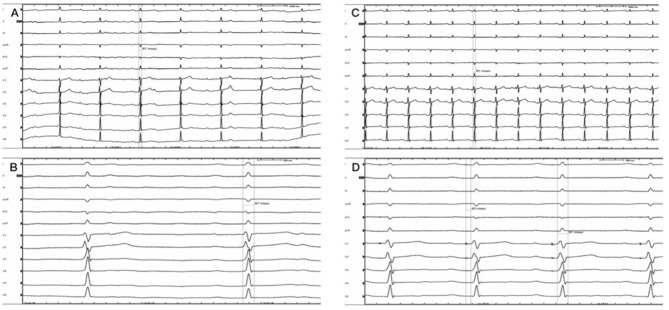
The comparison between pre-procedure and postoperative. (**A**) Preoperative electrocardiogram with paper speed of 25 mm/s. (**B**) Preoperative electrocardiogram with paper speed of 100 mm/s. (**C**) Postoperative electrocardiogram with paper speed of 25 mm/s. (**D**) Postoperative electrocardiogram with paper speed of 100 mm/s.

*The KODEX group* Before the procedure, multiple anisotropic fields were induced by the external dielectric sensors attached to the body surface of the patient. After local anesthesia and the puncture of the right femoral vein, the quadrupole catheter was advanced into the right atrium (RA). Then the KODEX-EPD system was connected to the catheter, which received the sophisticated electrical field information transmitted from the catheter. With the guidance of KODEX-EPD system, based on the electrical field information, the 3D cardiac anatomical image was calculated, the 3D cardiac anatomical image was constructed, and the images of superior vena cava (SVC), inferior vena cava (IVC), right atrium (RA), tricuspid valve (TVA), right atrial appendage (RAA) and right ventricular (RV) were visualized by roving the catheter within the cardiac chamber without fluoroscopy. The process can be done in less than five minutes. Under guidance of the KODEX-EPD system, His Bundle potential was mapped and marked above the TVA location and the posterior edge of the ventricular septum. (Fig. 2). Then the quadrupole catheter was removed, the ventricular lead (Model 3830, Medtronic, USA) was delivered with the guidance of Mode C315 His bundle sheath (Medtronic), the spiral tip of the lead’s cathode was exposed about 0.5 cm in low tension of His bundle sheath, which was shown clearly in the 3D model of KODEX-EPD for guiding electrode positioning. At the same time, the Panorama View (‘PANO View’) provided by the system transformed the 3D cardiac structure into a virtual two-dimensional (2D) panoramic picture and showed the endocardial surface. This view enhanced the perception of the lead position and orientation in the heart. The lead was adjusted slightly and fixed in the HB region finally (Fig. 2, the shown region of yellow point in 3D model). The pacing parameters and electrocardiogram were recorded (Figs. 3, 4, 5). Similarly, without the use of X-ray, anatrial lead was placed in the RAA based on the anatomical images.

### Data collection at follow-up

All patients were routinely followed-up after the procedure by outpatient clinic. Pacemaker function was assessed on the next day post-implantation and subsequently 6-months later at the pacing clinic. The pacing parameters including capture threshold, atrial/ventricular sensing and impedance were recorded during the procedure and at 6-months follow-up. Baseline data, such as demographic characteristics, implantation indications, and electrocardiographic measurements were collected at enrollment. Procedure-related data including total procedure time, fluoroscopic time and fluoroscopic dose were collected.

### Statistical methods

SPSS 26.0 statistical software was used for analysis. Before data analysis, Shapiro–Wilk test was used to statistically check the normality of the data. The data with normal distribution were expressed by Mean ± SD. Discrete data is expressed in percentage (%). An independent two sample t-test was used to compare the differences between two groups if the data were normally distributed, while u-test was performed for data that were not normally distributed. Fisher’s exact probabilities test was used for categorical variables to determine the differences between groups. A two-sided *p* < 0.05 was considered statistically significant.

## Results

### Baseline characteristics

There were 40 patients were enrolled in this study, including 27 males, with an average age of 60.9 ± 15.7 years. The KODEX group included twelve patients with atrioventricular block (including second degree type II block, third degree block and high degree block), seven patients with sinus node dysfunction (including sinus bradycardia, sinus cardiac arrest) and one patient with dilated cardiomyopathy. The standard group included fifteen patients with atrioventricular block (including second degree type II block, third degree block and high degree block), five patients with sinus node dysfunction (including sinus bradycardia and sinus cardiac arrest). The baseline characteristics between the two groups which included left ventricular ejection fraction (LVEF) and left ventricular end-diastolic dimension (LVEDD) were not statistically significant different (*p* > 0.05) (Table 1).

**Table 1:** Baseline characteristics between the two groups.

	Standard group (n = 20)	KODEX group (n = 20)	*P*-value
Demographics			
Age (years)	62.3 ± 18.9	59.6 ± 12.2	0.58
Male, n (%)	13 (65%)	14 (70%)	0.28
BMI	25.3 ± 3.8	25.7 ± 3.8	0.74
Indications			
SND, n (%)	5 (25%)	17(85%)	
AVB, n (%)	15 (75%)	12 (60%)	
DCM, n (%)	0 (0%)	1 (5%)	
Baseline ECG			
LBBB, n (%)	0 (0%)	0 (0%)	1.00
RBBB, n (%)	0 (0%)	1 (5%)	1.00
QRS duration (ms)	130.1 ± 8.7	131.2 ± 12.5	0.96
Echocardiography			
LVEF (%)	58.3 ± 6.9	57.9 ± 9.1	0.54
LVEDD (mm)	51.8 ± 6.5	51.6 ± 6.5	0.22

*AVB* atrioventricular block, *BMI* body mass index, *DCM* dilated cardiomyopathy, *ECG* electrocardiogram, *LBBB* left bundle branch block, *LVEDD* left ventricular end-diastolic diameter, *LVEF* left ventricular ejection fraction, *RBBB* right bundle branch block, *SND* sinus node dysfunction.

### Grouping and comparison

As shown in Table 2, there were no statistically significant differences in atrial impedance, atrial sensing, ventricular sensing, atrial pacing threshold and ventricular pacing threshold between the standard group and KODEX group (643.0 ± 98.8 vs. 591.5 ± 92.1 Ω, 2.9 ± 0.8 vs. 2.5 ± 0.8 mV, 12.8 ± 2.4 vs. 13.3 ± 3.3 mV, 1.0 ± 0.2 vs. 1.0 ± 0.1 V/0.4 ms, 1.0 ± 0.2 vs. 0.9 ± 0.1 V/0.4 ms, *p* > 0.05). Compared to the standard group, the KODEX group had statistically significant difference in ventricular impedance (640.0 ± 80.3 vs. 702.0 ± 86.1 Ω, i < 0.05). Because the operation requires repeated twisting, the longer the operation time, the higher the local tissue edema could be, which affected the result of impedance.

**Table 2:** Intraoperative related indicators.

	Standard group(n = 20)	KODEX group (n = 20)	*P*-value
Successful HBP	20 (100%)	20 (100%)	1.00
Procedure date			
Procedure time (min)	78.2 ± 25.1	63.7 ± 9.3	0.33
Fluoroscopy time (min)	19.3 ± 5.1	3.8 ± 0.5	< 0.05
Exposure dose (mGy)	120.2 ± 38.3	23.6 ± 5.4	< 0.05
Impedance (Ω)			
Atrial	591.5 ± 92.1	643.0 ± 98.8	0.09
Ventricle	702.0 ± 86.1	640.0 ± 80.3	< 0.05
Sensing (mV)			
Atrial	2.5 ± 0.8	2.9 ± 0.8	0.08
Ventricle	13.3 ± 3.3	12.8 ± 2.4	0.63
Threshold (V/0.4 ms)			
Atrial	1.0 ± 0.1	1.0 ± 0.2	0.81
Ventricle	0.9 ± 0.1	1.0 ± 0.2	0.63

### Procedure characteristics

As shown in Table 2, there were no significant differences in total procedure time between the standard group and KODEX group (63.7 ± 9.3 vs. 78.2 ± 25.1, *p* = 0.33). Procedure time was not statistically significant because of the relatively small sample size (N = 20 in each group) and the large variability of operation time in the control group. Compared to the standard group, the KODEX group had a significantly lower intraoperative fluoroscopy time (3.8 ± 0.5 vs. 19.3 ± 5.1 min, *p* < 0.05) and lower conventional fluoroscopy dose (23.6 ± 5.4 vs. 120.2 ± 38.3 mGy, *p* < 0.05). This was due to the three-dimensional reconstruction of cardiac anatomy by the KODEX system during procedure.

### Postoperative follow-up

All patients were followed up routinely, at one month, three months and six months after the procedure.

Intracardiac electrogram amplitude, impedance and pacing threshold of the HBP lead were shown in the device’s programmer system which were in the expected range. However, there will be a subacute period after operation, and the threshold of the subacute period will have a transient increase, which is considered to be related to transient postoperative local tissue edema. After one month, the threshold will tend to be stable. Forty (40) patients were followed up routinely, the average follow-up time was (5.9 ± 0.3) months. As shown in Table 3, there was no statistical significant difference in pacing parameters between the two groups at 6 months after procedure (*p* > 0.05). At 6 months follow-up, the preoperative LVEF and LVEDD compared with the postoperative values were not statistically significant different (*p* > 0.05). There were no procedure related complications in both groups, such as lead dislodge.

**Table 3:** Comparison of postoperative 6-month follow-up parameters.

	Standard group(n = 20)	KODEX group(n = 20)	*P*-value
Mean follow-up time (month)	5.9 ± 0.4	5.9 ± 0.3	0.81
Impedance (Ω)			
Atrial	501.1 ± 61.7	511.8 ± 56.7	0.57
Ventricle	603.1 ± 137.1	583.2 ± 128.8	0.63
Sensing (mV)			
Atrial	3.0 ± 0.7	2.9 ± 0.8	0.90
Ventricle	14.1 ± 3.1	13.4 ± 2.1	0.41
Threshold (V/0.4 ms)			
Atrial	0.6 ± 0.2	0.7 ± 0.2	0.44
Ventricle	0.9 ± 0.3	0.9 ± 0.3	1.00
Echocardiography			
LVEF (%)	57.7 ± 7.2	60.3 ± 5.3	0.20
LVEDD(mm)	51.4 ± 3.0	50.6 ± 4.8	0.61

*LVEDD* left ventricular end-diastolic diameter, *LVEF* left ventricular ejection fraction.

## Discussion

In recent years, physiological pacing is a major area of interest in cardiac pacing technology, in particular HBP is a discussion hotspot, as HBP can realize physiological synchronization, reverse ventricular remodeling and improve cardiac function, providing clinically relevant benefits for the pacemaker patient population. With the improvement of the pacing lead and the further optimization of the placement methods, there is a promising future for HBP. From previous studies it can be concluded that HBP achieves beneficial effects, that HBP can maintain cardiac function and significantly improve LVEF in patients with systolic dysfunction and cardiac failure^11^. As accurate positioning of the lead typically requires significant use of X-ray fluoroscopy, limiting its adoption and widespread use. In this study, the KODEX system was introduced to guide traditional pacing lead implantation, which could directly, visually, and accurately guide the HB region and improve the workflow of the procedure, especially with respect to fluoroscopy time and dose.

Previously, the workflow of HBP procedure was carried out using X-ray fluoroscopy, which has the risk of causing radiation damage to both patients and physicians as cancer risk are highly correlated with the X-ray dose^12^. With the guidance of the KODEX system in a HBP procedure, part of fluoroscopy exposure can be avoided, the X-ray exposure time and exposure dose can be reduced. Simultaneously, in clinical practice, the KODEX-EPD mapping system is also used in radiofrequency ablation and cryoballoon ablation (CBA). Pulmonary vein (PV) isolation using cryoballoon (CB) catheter has usually been characterized by high radiation and contrast media exposure^13^. Studies demonstrate that the KODEX-EPD system can guide CBA in a way that requires no x-rays and that its PV occlusion tool can provide real-time feedback during the procedure and the visualizable sheaths can effectively reduce fluoroscopic exposure, thus supporting the realization of zero or minimal fluoroscopic AF ablation^14–17^. Therefore, the KODEX-EPD 3D electroanatomical mapping system is a tool which can realize imaging the cardiac structure near zero X-ray.

The KODEX-EPD system provides two different ways to visualize the structure of cardiac anatomy. Firstly, 3D anatomy images offer a more conventional display of the cardiac chambers that allows for free rotation. Previous studies demonstrated the imaging accuracy of the KODEX-EPD system. The distance accuracy of the system is 1.08 ± 0.11 mm and the accuracy of the catheter position is 0.35 mm^12^. About the measurement of the pulmonary vein diameter, which was highly consistent with the measurement results of the pulmonary vein angiography as reference and the golden standard^18^, and the anatomic variation of the pulmonary vein was shown, which was not so much visible in preoperative cardiac CT^19^. Compared with the NavX, the KODEX-EPD system allows to build the anatomy with a new technology based on dielectric-sensing acquiring anatomy 1 cm ahead of the electrodes; the system recognizes the bending of the electric field due to blood vessel steep field gradients. A recent study suggested that KODEX-EPD was comparable to the Carto system in atrial modeling in real time by measuring the pulmonary vein diameter and the left atrial radial line. The KODEX-EPD system's original Panorama View (PANO View) of the anatomical structure of the cardiac chambers also could unfold the entire cardiac chamber for visualization of the endocardial surface, which shortens the learning curve. Furthermore, PANO View improved both the success rate and efficiency of the atrioventricular nodal reentrant tachycardia (AVNRT) ablation^12^. In this study, the 3D model was combined with PANO View which can be helpful to accurately locate the Koch triangle position, including the HB region.

In our study, we compared the KODEX-EPD system with conventional two-dimensional X-ray fluoroscopy and combined this information with intraoperative and follow-up data. We found that in comparison to conventional two-dimensional X-ray fluoroscopy, KODEX-EPD could significantly reduce fluoroscopy time and dose while maintaining similar pacing performance parameters.

During the 6-month follow-up, there were no procedure related complications in both groups. It can be shown that conduction system pacing implantation under the guidance of the KODEX-EPD system was feasible, safe and effective. Our results are aligned with those of earlier studies. A case report showed that the KODEX-EPD system could successfully guide CRT implantation in a 72-year-old female with a life-threatening contrast dye allergy^20^. Hua et al.^21^ successfully used KODEX-EPD in guiding pacemaker implantation in a patient with sick sinus syndrome. He subsequently completed ten cases of HBP with the guidance of the KODEX-EPD system^9^. The study showed that KODEX-EPD could facilitate HBP implantation with a similar success rate compared to the standard approach, and the fluoroscopic time and dose were significantly lower in the KODEX group, while the procedural time was not prolonged^9^.

This technology addresses the idea of “green electrophysiology,” and it offers a promising starting point for further investigation into precise pacing electrophysiology and patient safety. For patients who can’t be exposed to X-ray, such as those who are pregnant, have hematological conditions, have malignant tumors, or have other conditions, the technology is used as an alternative procedure method.

## Limitations

This study has several limitations. First, it is a single center study with a small sample size. However, this study is the first to examine the feasibility and safety of KODEX-EPD-guided HBP with a mid-term follow-up. Multicenter studies with a larger population are needed to further evaluate other potential merits with the KODEX-EPD system.

## Conclusion

This study demonstrates that conduction system pacing is feasible under guidance of the KODEX-EPD system, thereby significantly reducing fluoroscopy time and dose during the procedure. Intraprocedural and mid-term follow-up data show safety and effectiveness. The KODEX-EPD system is a novel near zero-fluoroscopic guidance tool which can be used to guide the implantation of conduction pacing leads.

## Data Availability

The data that support the findings of this study are available from the corresponding author upon reasonable request.
